# Why Do Hubs Tend to Be Essential in Protein Networks?

**DOI:** 10.1371/journal.pgen.0020088

**Published:** 2006-06-02

**Authors:** Xionglei He, Jianzhi Zhang

**Affiliations:** Department of Ecology and Evolutionary Biology, University of Michigan, Ann Arbor, Michigan, United States of America; National Institute of Genetics, Japan

## Abstract

The protein–protein interaction (PPI) network has a small number of highly connected protein nodes (known as hubs) and many poorly connected nodes. Genome-wide studies show that deletion of a hub protein is more likely to be lethal than deletion of a non-hub protein, a phenomenon known as the centrality-lethality rule. This rule is widely believed to reflect the special importance of hubs in organizing the network, which in turn suggests the biological significance of network architectures, a key notion of systems biology. Despite the popularity of this explanation, the underlying cause of the centrality-lethality rule has never been critically examined. We here propose the concept of essential PPIs, which are PPIs that are indispensable for the survival or reproduction of an organism. Our network analysis suggests that the centrality-lethality rule is unrelated to the network architecture, but is explained by the simple fact that hubs have large numbers of PPIs, therefore high probabilities of engaging in essential PPIs. We estimate that ~ 3% of PPIs are essential in the yeast, accounting for ~ 43% of essential genes. As expected, essential PPIs are evolutionarily more conserved than nonessential PPIs. Considering the role of essential PPIs in determining gene essentiality, we find the yeast PPI network functionally more robust than random networks, yet far less robust than the potential optimum. These and other findings provide new perspectives on the biological relevance of network structure and robustness.

## Synopsis

Proteins and their interactions form a protein–protein interaction network, where the proteins are the nodes and the interactions are the edges. Genomic studies show that deleting a highly connected protein node (hub) is more likely to be lethal to an organism than deleting a lowly connected node (non-hub), a phenomenon known as the centrality-lethality rule. Because hubs are more important than non-hubs in organizing the global network structure, the centrality-lethality rule is widely believed to reflect the significance of network architecture in determining network function, a key notion of systems biology. In this work, the authors proposed a small fraction of randomly distributed essential interactions, each of which is lethal to an organism when disrupted. Under this scenario, a hub is more likely to be essential than a non-hub simply because the hub has more interactions and thus a higher chance to engage in an essential interaction. Hence, the centrality-lethality rule is explained without the involvement of network architecture. Using yeast data, the authors provided empirical evidence supporting their hypothesis. Their proposal and results challenge a prevailing view in systems biology and provide a new perspective on the role of network structures in biology.

## Introduction

A network is composed of multiple nodes connected by edges. Most complex networks are scale-free, with a power-law distribution of the number of edges per node, or node connectivity [1,2]. That is, a scale-free network contains a small number of highly connected nodes (hubs) and a large number of poorly connected nodes (non-hubs). The relative importance of a node in a network is often measured by the magnitude of changes in network structure caused by the removal of the node. More accurately, such a measure should be termed the structural importance of a node. For instance, computational analysis shows that removing hubs increases the proportion of unreachable pairs of nodes and the mean shortest path length between all pairs of reachable nodes in the network (i.e., network diameter) more than removing non-hubs [3]. Hence, hubs are more important than non-hubs to the maintenance of the global network structure. In biomolecular networks, where genes or proteins are nodes and molecular interactions are edges, the importance of a node can also be measured by the magnitude of changes in network function or organismal fitness caused by the removal of the node. Such a measure may be called the functional importance of a node. For example, genome-wide gene deletion studies show that a small faction of genes in a genome are indispensable to the survival or reproduction of an organism [4,5]; these genes are referred to as essential genes. It was found that in the scale-free protein–protein interaction (PPI) network [6–8], hubs tend to be essential [6]. This phenomenon has been observed in the yeast, nematode, and fly [9–11] and is commonly referred to as the centrality-lethality rule [6]. Using the terms described above, the centrality-lethality rule indicates a correlation between a node's structural importance in the PPI network and its functional importance. Without critical analysis, this correlation has been widely interpreted as a causal relationship. That is, functional importance of a node is thought to arise from its structural importance in the network [6,7,9,10]. If true, this interpretation suggests a biological significance of network structures and hence is fundamental to systems biology. We here challenge this view by proposing an alternative explanation of the centrality-lethality rule that does not invoke the network architecture. We then evaluate the new explanation with empirical data and demonstrate that the prevailing interpretation of the centrality-lethality rule is unlikely to be correct.

## Results/Discussion

### An Alternative Explanation of the Centrality-Lethality Rule Based on Essential PPIs

The current analysis of PPI networks treats all edges equally. But in reality, some PPIs are more important than others. This consideration would be particularly meaningful if there are PPIs that are essential (indispensable) to the survival or reproduction of an organism. An essential interaction between two proteins makes both proteins essential, because the removal of either protein causes lethality or infertility due to the disruption of the interaction. Empirical data indicate the existence of essential PPIs. For example, yeast proteins SPT16 and POB3 are both essential and they form heterodimers that function in DNA replication; genetic studies showed that their interaction is critical for this function [12]. Essential PPIs can potentially explain the centrality-lethality rule, because proteins with more PPIs have a greater probability to engage in at least one essential PPI, thus having a higher chance to be essential. Note that the network architecture is not invoked in this explanation.

### Evaluation of the Number of Essential PPIs and Their Contribution of Gene Essentiality

It is difficult to identify essential PPIs experimentally at the genomic scale, because the identification requires the demonstration that disrupting the interaction between two essential proteins without affecting any other aspects of the protein functions causes lethality or infertility. Here we use a computational approach to evaluate the prevalence of essential PPIs and the contribution of essential PPIs to gene essentiality at the genomic level. Our analysis focuses on the yeast Saccharomyces cerevisiae because both the PPI and gene essentiality data are most complete in this species.

**Figure 1:**
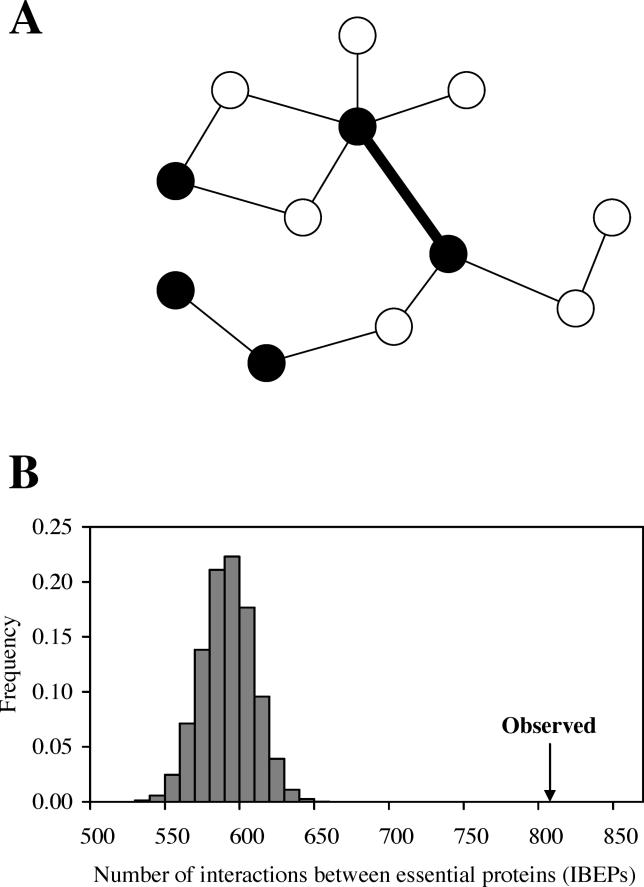
Essential Edges (Interactions) in PPI Networks (A) A hypothetical PPI network of 12 proteins. Black and white nodes refer to essential and nonessential proteins, respectively. Thick and thin edges depict essential and nonessential interactions, respectively. Proteins linked by an essential interaction must be essential, whereas an interaction between essential proteins (IBEP) may or may not be essential. (B) More IBEPs in the yeast PPI network than in randomly rewired networks. “Observed” indicates the observed number (807) of IBEPs in the real network. The gray bars show the distribution of the number *(m)* of IBEPs in 10,000 randomly rewired networks.

We built our yeast PPI network, in which 4,126 protein nodes are linked by 7,356 edges. The PPI data we used were compiled manually by the Comprehensive Yeast Genome Database [13] from the literature and published large-scale experiments. As mentioned, two proteins forming an essential PPI must be essential (Figure 1A). On the contrary, interactions between essential proteins (IBEPs) may or may not be essential, because the essentiality of a protein can be due to factors other than essential PPIs (Figure 1A). This feature allows us to estimate the number of essential PPIs in a network, as the number of IBEPs increases with the number of essential PPIs. There are 807 IBEPs in our network. We generated a control network by randomly rewiring all edges of the real network while keeping the node connectivity *(k)* unchanged for every node. By repeating this procedure 10,000 times, we obtained the distribution of the number *(m)* of IBEPs in randomly rewired networks (Figure 1B). The mean of *m* is 592.6. None of the 10,000 *m* values is greater than the number of IBEPs in the real network, strongly suggesting an excess of IBEPs in the real network (*p* < 0.0001). This excess is also evident in different datasets of yeast PPIs and in nematode PPIs [14,15] (see below and Figures S1 and S2). Under the assumption that the excess of IBEPs is entirely caused by essential PPIs, we estimate that α = (807–592.6)/7356 = 2.92% of interactions in the yeast PPI network are essential. The standard error of α is 0.23%. Here we used random rewiring to estimate α because there are no easy ways to calculate α analytically unless self-interactions are allowed.

In our network, 836 proteins, or 20.3% of all nodes, are essential. In addition to essential PPIs, there are other factors (e.g., protein-DNA interaction) that could render a protein essential. Let β be the probability that a node becomes essential by these other factors. To estimate β, we first remove the information of gene essentiality in the yeast PPI network. We then randomly assign 807-*m* essential edges to this network, where *m* is randomly drawn from its distribution in Figure 1B. Note that 807-*m* is the estimated number of essential edges. Nodes having essential edges are marked essential. Next, we mimic the influence of the other factors that cause gene essentiality by randomly marking nodes as essential, until the total number of essential nodes in the network becomes 836. Repeating this procedure 10,000 times, we estimate that the essential PPIs render 8.7% of nodes essential, while the other factors render β = 12.64% of nodes essential. The standard error of β is 0.63%. Note that some nodes (1.1%) are affected by both essential PPIs and the other factors. Approximately 43% (8.7%/20.3%) of protein essentiality is attributable to essential interactions in the PPI network.

Our estimates of α and β may be biased by several factors. First, some features of the yeast PPI network could have been distorted by random rewiring, thus affecting the estimation of α. For example, it is known that links between highly connected nodes are suppressed in PPI networks [16,17]. Because highly connected nodes tend to be essential [6,9–11], the suppression reduces the number of IBEPs. Hence, if this suppression was not accounted for in our rewiring, we may have overestimated *m,* and consequently underestimated α. However, this bias is probably small, as the suppression appears to be largely limited to nonessential proteins [14]. Second, the quality of the PPI data can affect the reliability of our estimates. In particular, transforming protein complex information to binary PPI data using either the “spoke” model (the bait is predicted to interact with all members of a complex) or the “matrix” model (all members of the complex are predicted to interact with all other members in the complex) [18] tends to generate extra IBEPs for large complexes, which would lead to an overestimation of α. However, our data do not include much of the protein complex information recently produced by high-throughput methods [19,20], and thus may be largely immune to this problem. Third, we assumed that the excess of observed IBEPs in the real network is entirely due to essential PPIs, while it may also be caused by other nonrandom features of the real network [14,15]. Finally, our estimation of β is based on the assumption that the other factors causing protein essentiality affect all nodes in the PPI network equally in a random manner.

**Figure 2:**
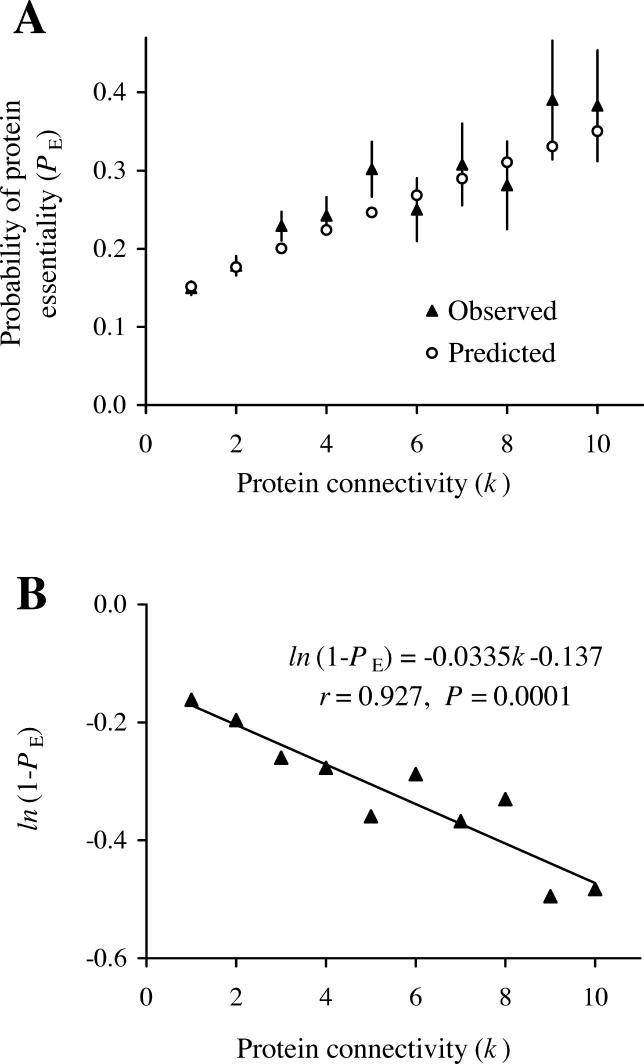
The Relationship between the Probability That a Protein Is Essential (*P*_E_) and the Connectivity *(k)* of the Protein (A) Observed and predicted *P*_E_ values. The observed values were estimated from the yeast PPI network and the predicted values were computed using Equation 1 with parameters α = 2.92% and β = 12.6%. Error bars show one standard (sampling) error of the observed values. (B) Linear regression between *ln*(1-*P*_E_) and *k*. Using Equation 2, we estimated from the regression that α = 3.29% and β = 12.8%. The 95% confidence interval for α is between 2.23%–4.35%. The 95% confidence interval for β is between 6.7%–18.6%. Proteins with *k* > 10 (~ 5% of all proteins) are not considered because of small sample sizes.


To examine these potential biases and to evaluate the reliability of our estimates, we conducted three tests. First, according to our analysis, factors other than essential PPIs render β = 12.6% of proteins essential. There are 1,952 yeast proteins that have no PPIs and thus are not included in our PPI network. Interestingly, 11.9% ± 0.8 % (233/1,952) of these proteins are essential, a number statistically indistinguishable from β (*p* > 0.4, chi-squares test). This congruence suggests that our estimate of β is reliable and the assumption of stochastically equal influences of these other factors on all nodes is acceptable. Second, because our estimation relied on simulated networks, we compared network features between simulated and real networks. In particular, node essentiality was randomly reassigned in the estimation of β, although the network structure was unaltered. We found that the frequency distribution of node connectivity is similar between the reassigned networks and the real network for both essential and nonessential nodes (Figure S3). This result suggests that the determination of node essentiality in the yeast PPI network is largely captured by our two-step procedure, which involves essential PPIs that are randomly distributed among edges and other essentiality-determining factors that are randomly distributed among nodes. The final and most critical evaluation of our estimates of α and β is to test whether protein essentiality can be predicted using these estimates. For a protein to be nonessential, two conditions must be satisfied. First, the protein has no essential PPI. Second, the protein is not affected by the other factors that cause essentiality. Thus, the probability (*P*_E_) that a protein with *k* PPIs is essential is:

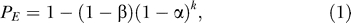
where α and β have been estimated earlier. Thus, *P*_E_ values can be predicted for each *k* using the above equation. Our observed *P*_E_ from the yeast PPI network matched well to the predicted *P*_E_ (Figure 2A). We did not compare *P*_E_ values for *k* > 10, because there are few nodes for each *k* value when *k* > 10. Equation 1 can be rewritten with natural logarithm as:

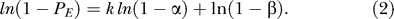


Equation 2 predicts that *ln*(1-*P*_E_) changes linearly with *k*. This linear relationship is confirmed for the yeast PPI network (correlation coefficient = 0.927, *p* = 0.0001; Figure 2B). We estimate that α = 3.29% and β = 12.8% from the slope and Y-intersect of the linear regression, respectively (Figure 2B). These estimates are not significantly different from our earlier estimates based on simulated networks (*p* > 0.5). Taken together, the three tests confirm that our estimates of α and β are reasonably good.

### Essential PPIs Are Evolutionarily More Conserved than Nonessential PPIs

It would be interesting to predict which PPIs are essential. But this prediction is naturally more difficult than estimating the percentage of PPIs that are essential, because of the scarcity of information for individual PPIs. Nonetheless, it is clear that only IBEPs can be essential. The probability that an IBEP is essential is (807–592.6)/807 = 0.27. Here 807 is the total number of IBEPs and (807–592.6) is the estimated number of essential interactions. If two interacting essential proteins do not interact with other essential proteins (observation *O*), the posterior probability that their interaction is essential (event *E*) can be derived from the Bayes theorem as:

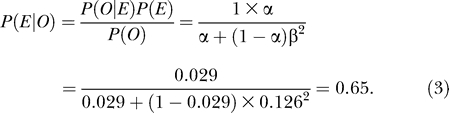



The yeast PPI network contains 38 such “probably essential” PPIs (see Table S1 for gene names and functions). Compared to nonessential PPIs, essential PPIs are expected to be more conserved in evolution due to their importance to the organismal survival and reproduction. To test this hypothesis, we assembled the PPI network of the fruit fly Drosophila melanogaster. There are 1,066 PPIs among the yeast proteins that have orthologs in the fruit fly, and 4.3% of these PPIs are conserved between the two species (Table 1 and Table S2). In comparison, 7.6% of IBEPs and 26.3% of probably essential PPIs are conserved between the species, confirming the prediction that essential PPIs are evolutionarily more conserved than nonessential PPIs (Table 1 and Table S2). Other than phylogenetic conservation, the 38 probably essential interactions do not show any special features. They are not apparently enriched in any functional categories, biological processes, or stable protein complexes. For example, 45% of the 38 probably essential interactions involve two proteins that appear in the same protein complexes, compared to 47% of the 748 other IBEPs (*p* > 0.5, χ^2^ test). It is possible that certain enrichment does exist, but is difficult to discern due to the small sample size.

**Table 1:**
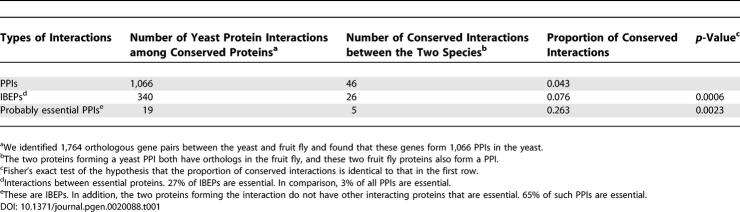
Conservation of Essential PPIs between the Yeast and Fruit Fly

### Essential PPIs Explain the Centrality-Lethality Rule

Our analysis of the yeast PPI network suggests that the centrality-lethality rule is due to the simple fact that highly connected nodes are involved in more PPIs than are poorly connected nodes, thus having greater probabilities of engaging in essential PPIs. One can see from Equation 1 that *P*_E_ is determined by only two factors. One of them is protein connectivity, arising solely from essential PPIs, whereas the other factor is independent of protein connectivity. The success of the equation in describing the empirical observations (Figure 2) and the congruence of the estimates of α and β obtained from two different approaches suggest that factors dependent on protein interactions, but unrelated to essential PPIs, are trivial, implying that gene essentiality is unlikely due to cumulative or pleiotropic effects at the PPI level. Furthermore, they suggest that among all structural features of the PPI network, protein connectivity is the sole determinant of protein essentiality, and that this determination is via essential PPIs. These results argue against the hypothesis that the centrality-lethality rule is attributable to the relative importance of hub proteins to the maintenance of the network architecture [6,7,9,10]. In support of our hypothesis, node centrality, as measured by betweenness or closeness, is not higher for essential nodes than for nonessential nodes in the yeast PPI network, after the control of node connectivity (Tables 2 and 3). Here, betweenness of a node is the proportion of shortest paths among all pairs of reachable nodes that go through the node, whereas closeness of a node is the mean shortest path length between the node and all reachable nodes in the network. Both betweenness and closeness measure the centrality of a node in the global network structure. Further support to our hypothesis comes from a recent analysis of the yeast PPI network, in which hubs were classified into two types according to the coexpression patterns between interacting proteins [21]. It was found that although removing one type of hub increases the network diameter more than removing the other type, the two types have similar essentiality [21,22].

**Table 2:**
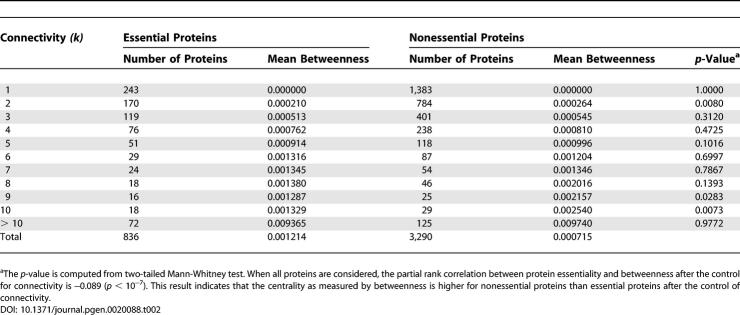
Normalized Betweenness of Essential and Nonessential Proteins in the Yeast PPI Network

**Table 3:**
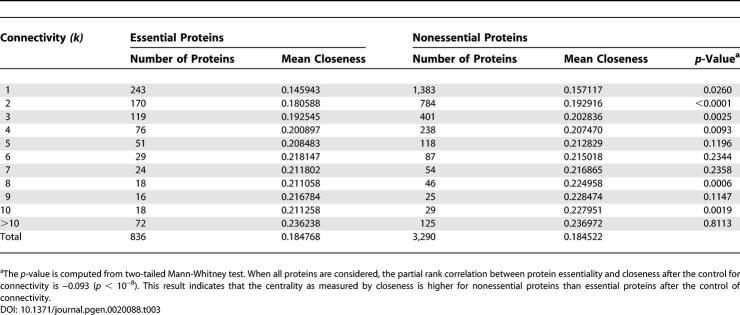
Normalized Closeness of Essential and Nonessential Proteins in the Yeast PPI Network

**Figure 3:**
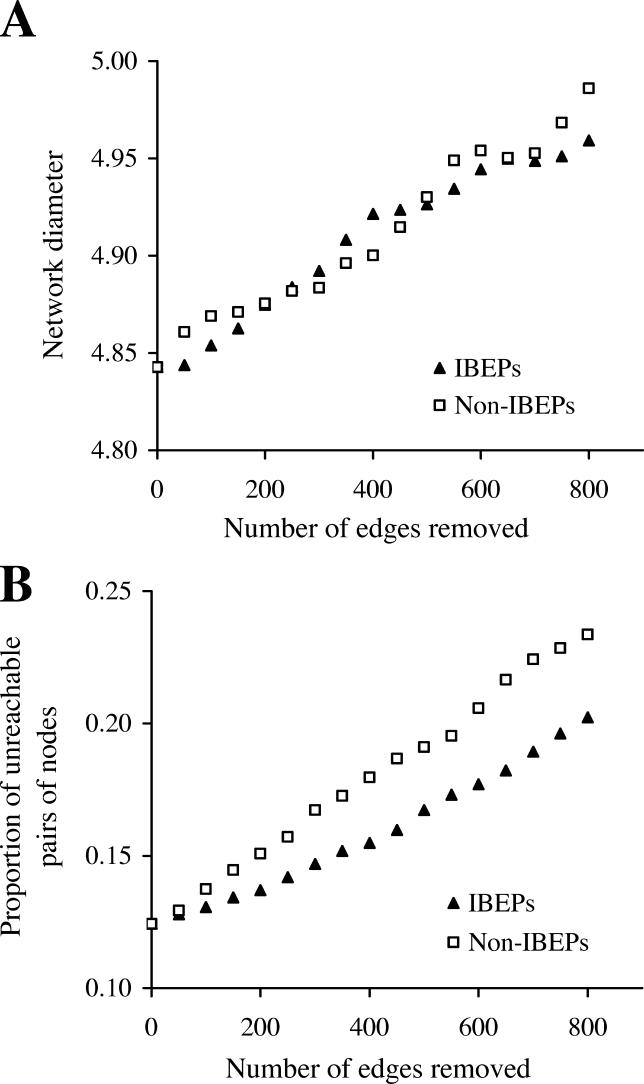
Effects of Random Removal of Edges on the Global Structure of the Yeast PPI Network (A) Effects on network diameter, which is the mean shortest path length among all reachable pairs of nodes in the network. (B) Effects on the proportion of unreachable pairs of nodes in the network. Note that the total number of IBEPs is 807 in the network.

One could argue that the essentiality of a PPI may be due to its special location in the network and that removing an essential PPI may disturb the network architecture more than removing a nonessential PPI. Unfortunately, it is unknown with certainty which PPIs are essential in the yeast network. Because only IBEPs may be essential, removing IBEPs is expected to increase the network diameter more than removing non-IBEPs, if essential PPIs are more important than nonessential PPIs in maintaining the network architecture. However, no such trend is found (Figure 3A). Moreover, removing IBEPs generates fewer unreachable pairs of nodes than removing non-IBEPs (Figure 3B). This is probably because IBEPs tend to occur between highly connected nodes, which are less affected than lowly connected nodes by the loss of an edge. Thus, there is no evidence that essential PPIs are more important than nonessential PPIs in maintaining the network architecture.

### The Yeast PPI Network Is Functionally More Robust than Random Networks

**Figure 4:**
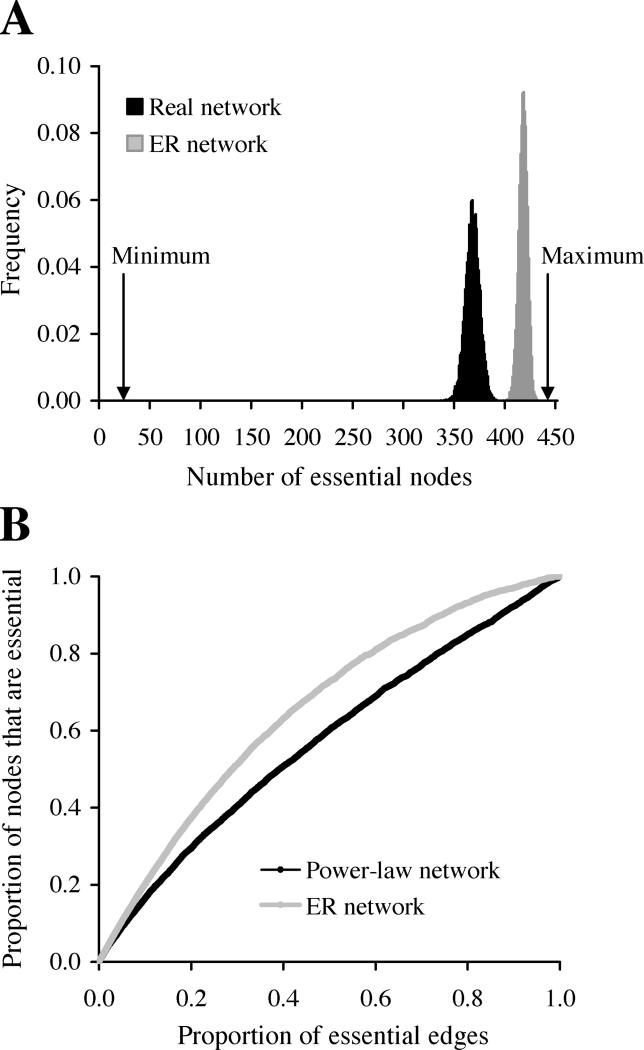
Robustness of PPI Networks (A) Numbers of essential nodes generated by 220 essential edges in various networks. Black and gray bars depict the distribution of the number of essential nodes from 10,000 replications of random assignments of 220 essential edges to the real yeast PPI network and simulated ER networks, respectively. An ER network has the same number of nodes and edges as in the real network, but the distribution of node connectivity follows a Poisson distribution. Also shown are the minimal and maximal numbers of essential nodes produced by 220 essential edges in any possible network that has the same numbers of nodes and edges as the yeast PPI network. The minimum is 22, because the number of edges among 22 nodes can be as high as 21 × 22/2 = 231 > 220. The maximum is 220 × 2 = 440. (B) Proportions of essential nodes generated by given numbers of essential edges in scale-free (power-law) and ER networks. Both networks contain 4,000 nodes and 4,352 edges. The scale-free network has its node connectivity following the power-law distribution *P(k)* ∝ *k* ^−γ^, where *P(k)* is the probability that a node has *k* edges. We used γ = 2.29, the same as in the real yeast PPI network (see Figure S4). The ER network has a connectivity distribution following the Poisson distribution with mean connectivity per node being 2.176. The result that more essential nodes are produced in ER networks than in scale-free networks by a given number of essential edges applies to other γ values (see Figure S5).

It is often said that scale-free networks are robust against random removals of nodes, because the majority of nodes are poorly connected, and they play relatively unimportant roles in organizing the global network structure [3]. Since in PPI networks the only factor determining protein essentiality is essential PPIs, it is possible to examine if the PPI network is structured in a particularly robust fashion. Based on the estimates of α from both network rewiring and linear regression, we assume that 220 edges (3% of all edges) in the yeast PPI network are essential. If we randomly assign 220 essential edges in the yeast PPI network, on average 368 nodes become essential (Figure 4A). If the connectivity distribution does not follow the power-law as in scale-free networks, but follows the Poisson distribution as in Erdös-Rényi (ER) random networks [23], on average 417 essential nodes would result from 220 essential edges (Figure 4A). In fact, the expected number of essential nodes generated by a given number of essential edges is always lower in scale-free networks than in ER networks (Figure 4B). This may suggest that the scale-free network is more robust than the ER network, even when we consider the underlying mechanism of node essentiality. Note that the above interpretation of network robustness is different from previous analyses. In previous investigations, robustness is measured in terms of network structure [3], but here it is measured by network function. We caution that the higher robustness of the scale-free yeast PPI network than ER networks does not imply that the robustness originated from natural selection for robustness [6]. More likely, robustness emerged as a byproduct of other evolutionary processes or contingencies. Furthermore, it is interesting to note that the yeast PPI network is far from the most robust network possible. For instance, one can design a network in which 220 essential edges link 22 essential nodes (Figure 4A). Obviously, evolution did not work in that way.

### Caveats

Our analysis is based on the PPI data in the Comprehensive Yeast Genome Database [13]. To examine whether our results are similar when different yeast PPI datasets are used, we tried two other datasets, one with many more nodes and edges [24] and the other with much fewer nodes and edges [21]. We found that using simulated networks and using linear regression gave similar estimates of α and β for a given dataset, although different datasets provided different estimates (Figures S1 and S2). These results are not unexpected, given that the three datasets we used vary greatly in the numbers of nodes and edges, mean connectivity, and proportion of essential nodes. These variations reflect different numbers of false-negative and false-positive data about protein essentiality and PPI among different datasets. The noise and incompleteness of the data could potentially undermine our ability to predict *P*_E_. However, as long as essential PPIs are randomly distributed among edges and the other essentiality-causing factors affect all nodes equally in a random fashion, our Equation 1 should work. In fact, the congruence between the estimates of α and β from simulated networks and regression analysis in each of the three datasets strongly suggest that our explanation of the cause of protein essentiality is largely correct. Under the assumption that false-negative and false-positive PPIs are randomly distributed in the network, false-negative PPIs do not affect α, because essential and nonessential PPIs are affected to the same extent. On the contrary, false-positive PPIs lead to an underestimation of α, because the number of essential PPIs is not affected, but the total number of PPIs is inflated. Both of these predictions were confirmed in a simulation where 50% of yeast PPIs were randomly removed or added. These findings suggest that α estimated from the dataset with minimal false-positive PPIs [21] may be most accurate. Nonetheless, this dataset contains fewer nodes than those of other datasets and therefore the estimated α may be applicable only to this subset of nodes. A recent study of pure high-throughput yeast two-hybrid data of PPIs showed a weaker centrality-lethality relationship than previously found from better corroborated data [25]. This result is expected because the pure high-throughput yeast two-hybrid data contain high proportions of false-positive PPIs, resulting in a lower α (e.g, 1.2% for Ito et al.'s data [26]) and consequently a weaker influence of *k* on *P*_E _ (see Equation 1).

It is well known that singleton genes are more likely to be essential than duplicate genes [4,27,28]. It is interesting to ask whether singletons are more likely than duplicates to engage in essential interactions. However, because singletons and duplicates do not form two separate PPI networks, it is impossible to estimate separate α values for them. Furthermore, potential functional compensations between duplicates could mask the true essentiality of a duplicate gene. That is, many nonessential duplicate genes may actually have essential PPIs. To avoid these problems, we classify genes into singletons and duplicates and examine their interaction partners, while ignoring the essentiality of these genes themselves. We found that yeast duplicate genes have on average 0.89 essential partners, significantly fewer than the expected number (0.94) estimated from 5,000 randomly rewired networks (*p* = 0.004). On the contrary, yeast singletons have on average 1.01 essential partners, significantly more than the expected number (0.94) estimated from randomly rewired networks (*p* = 0.002). This analysis suggests that essential PPIs potentially contribute to the higher essentiality of singletons than duplicates, supporting the view that singleton genes are intrinsically more important than duplicate genes [29].

### Implications

In biological networks as well as in other networks, different edges may be of different levels of importance. Treating these edges in a quantitatively or qualitatively different way may reveal previously unknown patterns and provide new insights. In this work, we propose the concept of essential protein interactions and demonstrate by computational network analysis that a large faction of gene essentiality is due to essential PPIs. It is important to stress that using essential PPIs to explain gene essentiality is not tautological, because the explanation provides a molecular understanding of why certain genes are essential and offers a conceptual framework for future experimental proofs. Logically, the next question is why essential PPIs are essential. We show that essential PPIs are no more likely to occupy central locations in the PPI network than nonessential PPIs. Thus, the essentiality of a PPI does not seem to be determined by network structures but rather by the particular functions of the interaction. Alternatively, the influence of the network architecture may be more subtle and thus require further scrutiny of larger and more accurate PPI data. Similarly, our results suggest a simpler explanation of the centrality-lethality rule that does not invoke the role of protein hubs in organizing the global network structure. Furthermore, our hypothesis quantitatively explains the centrality-lethality rule, whereas the network architecture hypothesis lacks such a quantitative model. Our finding appears to argue against the biological significance of the PPI network architecture. However, it should be pointed out that although gene essentiality is an important phenomenon because it determines organismal survival and reproduction, the significance of the network architecture may lie in other aspects of the cellular life that have yet to be explored. Furthermore, our analysis focuses on PPI networks, and it is unclear whether our results extend to other biomolecular networks. Therefore, the role of network architecture in biology cannot and should not be dismissed at this time. Rather, more studies are needed in the nascent field of systems biology to address such important questions as the biological meaning and evolutionary origin of the architecture and robustness of biological networks [7,30–32].

## Materials and Methods

The yeast PPI data were downloaded from ftp://ftpmips.gsf.de/yeast/PPI. Although self- interactions may contain important biological information, they were not considered in our analysis, mainly because our approach of using IBEPs to infer essential interactions would not work for self-interactions. Because the centrality-lethality rule is observed when self-interactions are excluded, our analysis should still be biologically meaningful. We also excluded from our analysis 43 interactions involving Ty elements and six involving mitochondrial genes, resulting in 7,356 non-redundant PPIs linking 4,126 yeast nuclear genes, of which 836 genes are essential. The mean connectivity per protein is 3.57. Yeast genes that were subject to single-gene deletion studies were listed in: http://www-deletion.stanford.edu/YDPM. Essential genes were listed in: http://www-sequence.stanford.edu/group/yeast_deletion_project/Essential_ORFs.txt. There were 162 genes in our protein network that lacked the essentiality information and were treated as nonessential in the analyses. This strategy might have rendered ~ 0.8% of the genes in our network misclassified in terms of gene essentiality. All of our results were virtually identical when these 162 genes were excluded from the protein network. Essential genes are those indispensable for the growth of yeasts in the YPD-rich media. This set of genes is apparently fundamental to the cellular processes of the yeast, although additional genes may become indispensable in adverse conditions [33]. Yeast stable protein complex dataset was downloaded from *Saccharomyces* Genome Database (ftp://genftp://genome-ftp.stanford.edu/pub/yeast/data_download/literature_curation/go_protein_complex_slim.tab), which contained 188 complexes comprising 1,226 genes.

Singleton genes and duplicate genes were defined by all-against-all BLASTP searches of yeast proteins, following [34]. Specifically, a gene was considered a singleton if there were no non-self hits at E-value = 0.1. A gene was considered a duplicate if it had at least one non-self hit at E-value = 10^−20^.

The fruit fly PPI network [35] included 4,579 proteins connected by 4,663 non-self high-confidence interactions. We conducted a genome-wide all-against-all BLASTP search (E-value cutoff = 10^−10^) between 5,773 yeast and 13,434 fruit fly proteins, which were downloaded from *Saccharomyces* Genome Database (http://www.yeastgenome.org) and ENSEMBL (http://www.ensembl.org), respectively. 1,764 reciprocal best hits were found, and they were considered as orthologous proteins between the two species. To control for the fact that essential genes tend to be evolutionarily conserved, we examined only those yeast PPIs for which both partners have orthologs in the fruit fly. The above 1,764 proteins form 1,066 PPIs in the yeast and 156 PPIs in the fruit fly.

Network parameters such as the diameter, closeness, and betweenness were calculated using the computer software Pajek, downloaded from: http://vlado.fmf.uni-lj.si/pub/networks/pajek. The node connectivity in our yeast PPI network can be approximated by a power-law distribution with the parameter γ = 2.29 (Figure S4). To simulate a scale-free (power-law) network with parameter γ (for Figure 4B), we first computed *P(k),* the expected frequency of nodes with *k* edges (*k* = 1, 2, 3, …), using *P(k)* = *ak* ^−γ^, where *a* is a constant determined by

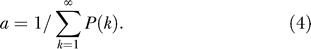
We then decided the connectivity of each of the 4,000 nodes in the network following the above *P(k)* distribution and randomly paired the nodes by considering the connectivity. When generating the corresponding ER network, we randomly paired the 4,000 nodes until the total number of edges reached that of the corresponding scale-free network.

## Supporting Information

Figure S1 Relationship between the Probability That a Protein Is Essential (*P*_E_) and the Connectivity *(k)* of the Protein in the Yeast PPI Network The yeast PPI information was downloaded from GRID (General Repository of Interaction Datasets) [24] at http://biodata.mshri.on.ca/yeast_grid/files/Full_Data_Files/interactions.txt. After excluding self-interactions and interactions involving Ty elements or mitochondrial genes, a total of 13,189 physical PPIs connecting 4,674 genes (including 972 essential genes) were obtained. (A) Observed and predicted *P*_E_ values. The observed values were estimated from the yeast PPI network. Error bars show one standard (sampling) error of the observed values. The predicted values were computed using Equation 1 with parameters α = 4.2% ± 0.2 % and β = 3.5% ± 0.8 %, which were estimated using rewired and essentiality-reassigned networks as described in the main text (5,000 replications). (B) Linear regression between *ln*(1-*P*_E_) and *k*. We estimated from the regression and Equation 2 that parameters α = 4.2% and β = 4.9%. Proteins with > 10 edges (~ 14% of all proteins) were not considered due to the paucity of data for each *k*.
(12 KB PDF)

Figure S2 Relationship between the Probability That a Protein Is Essential (*P*_E_) and the Connectivity *(k)* of the Protein in the Yeast PPI Network The yeast PPI information compiled by Han and colleagues [21] was downloaded from: http://www.nature.com/nature/journal/v430/n6995/suppinfo/nature02555.html. There are 2.493 interactions among 1,379 genes (including 530 essential genes). (A) Observed and predicted *P*_E_ values. The observed values were estimated from the yeast PPI network. Error bars show one standard (sampling) error of the observed values. The predicted values were computed using Equation 1 with parameters α = 7.4% ± 0.5 % and β = 21.8% ± 1.4 %, which were estimated using rewired and essentiality-reassigned networks as described in the main text (10,000 replications). (B) Linear regression between *ln*(1-*P*_E_) and *k*. We estimated from the regression and Equation 1 that parameters α = 7.3% and β = 24.9%. Because of the paucity of proteins with high connectivity, those with six and seven edges were considered together and counted as 6.5 edges, and those with eight and nine edges were considered together as 8.5 edges. Proteins with ≥ 10 edges (~ 8% of all proteins) were not considered due to the paucity of data for each *k*.
(11 KB PDF)

Figure S3 Similarity in Node Connectivity between the Yeast PPI Network and Simulated Networks for (A) Essential and (B) Nonessential Nodes To construct the simulated networks, we removed the node essentiality information from the real network and then reassigned node essentiality in a two-step random fashion (see main text). The mean frequencies are shown for 10,000 simulated networks.
(10 KB PDF)

Figure S4 Frequency Distribution of Connectivity *(k)* per Protein in the Yeast PPI Network Follows the Power-Law *P(k)* ∝ *k* ^−2.29^
The network contains 4,126 nodes connected by 7,356 edges.
(41 KB PDF)

Figure S5 Proportions of Essential Nodes Generated by Given Numbers of Essential Edges in Scale-Free and ER Networks The scale-free network has the node connectivity following the power-law distribution, and the ER network has the node connectivity following the Poisson distribution. (A) Comparison between the power-law network with γ = 2 and the ER network. Both networks contain 4,000 nodes and 5,995 edges and are randomly generated following the respective connectivity distributions. (B) Comparison between the power-law network with γ = 2.5 and the ER network. Both networks have 4,000 nodes and 3,620 edges.
(130 KB PDF)

Table S1 Probably Essential PPIs in the Yeast (53 KB PDF)

Table S2 Conserved PPIs between the Yeast and Fruit Fly (70 KB PDF)

## Abbreviations

ER: Erdös-Rényi
IBEP: interaction between essential proteins
PPI: protein–protein interaction
