# Supplementary material for: Why Do Hubs Tend to Be Essential in Protein Networks?
Source: PLoS Genet. 2006 Jun 2;2(6):e88. doi: 10.1371/journal.pgen.0020088 (PMC1473040; doi:10.1371/journal.pgen.0020088)
Supplement: Supplementary file 1 [file pgen.0020088.sg001.pdf]

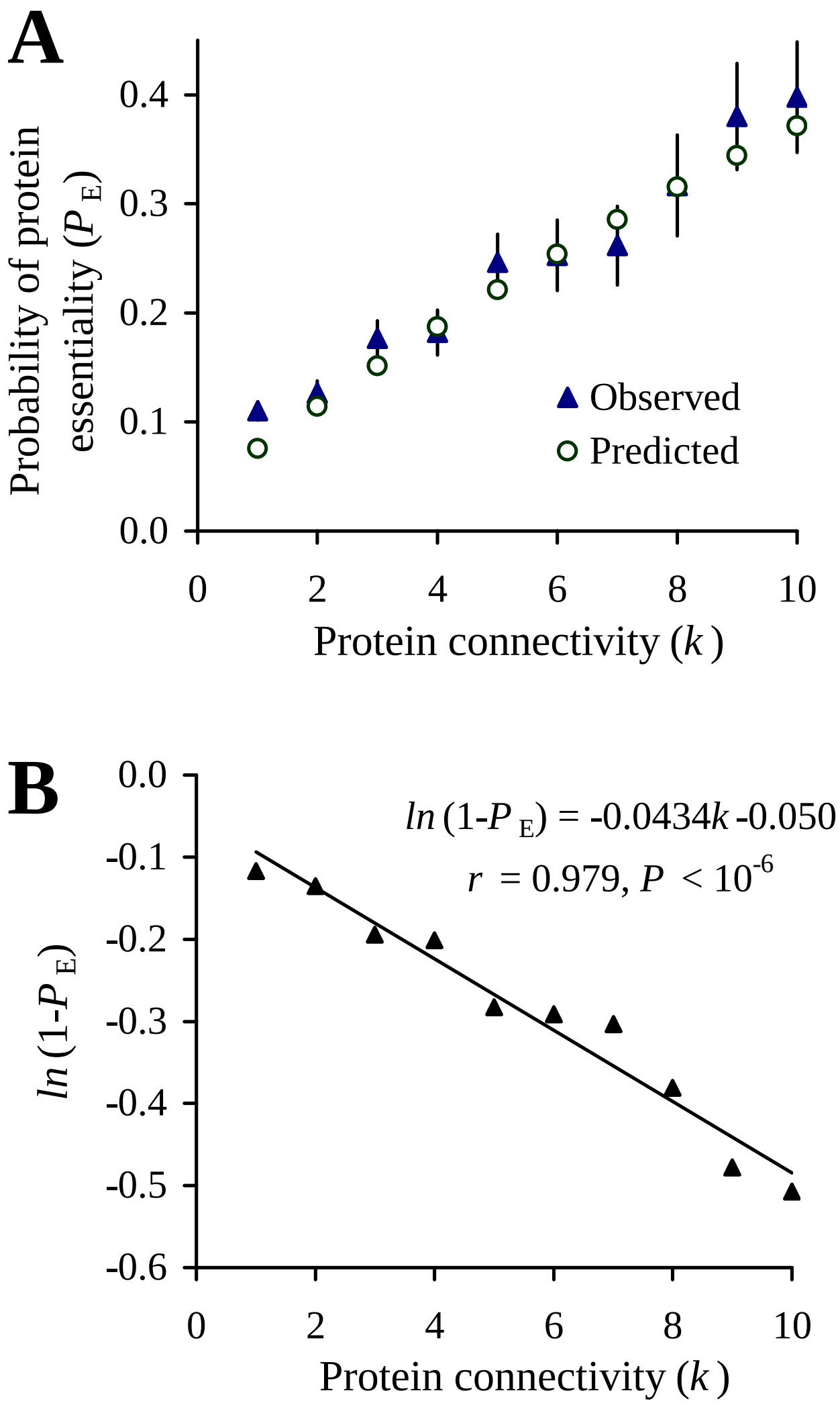
A
Probability of protein essentiality (P E)
0.0
0.1
0.2
0.3
0.4
Observed
Predicted
0
2
4
6
8
10
Protein connectivity (k)
B
ln (1-P E) = -0.0434k -0.050
r = 0.979, P < 10-6
ln (1-P E)
0.0
-0.1
-0.2
-0.3
-0.4
-0.5
-0.6
0
2
4
6
8
10
Protein connectivity (k)

Figure S1
