# Supplementary material for: Why Do Hubs Tend to Be Essential in Protein Networks?
Source: PLoS Genet. 2006 Jun 2;2(6):e88. doi: 10.1371/journal.pgen.0020088 (PMC1473040; doi:10.1371/journal.pgen.0020088)
Supplement: Supplementary file 2 [file pgen.0020088.sg002.pdf]

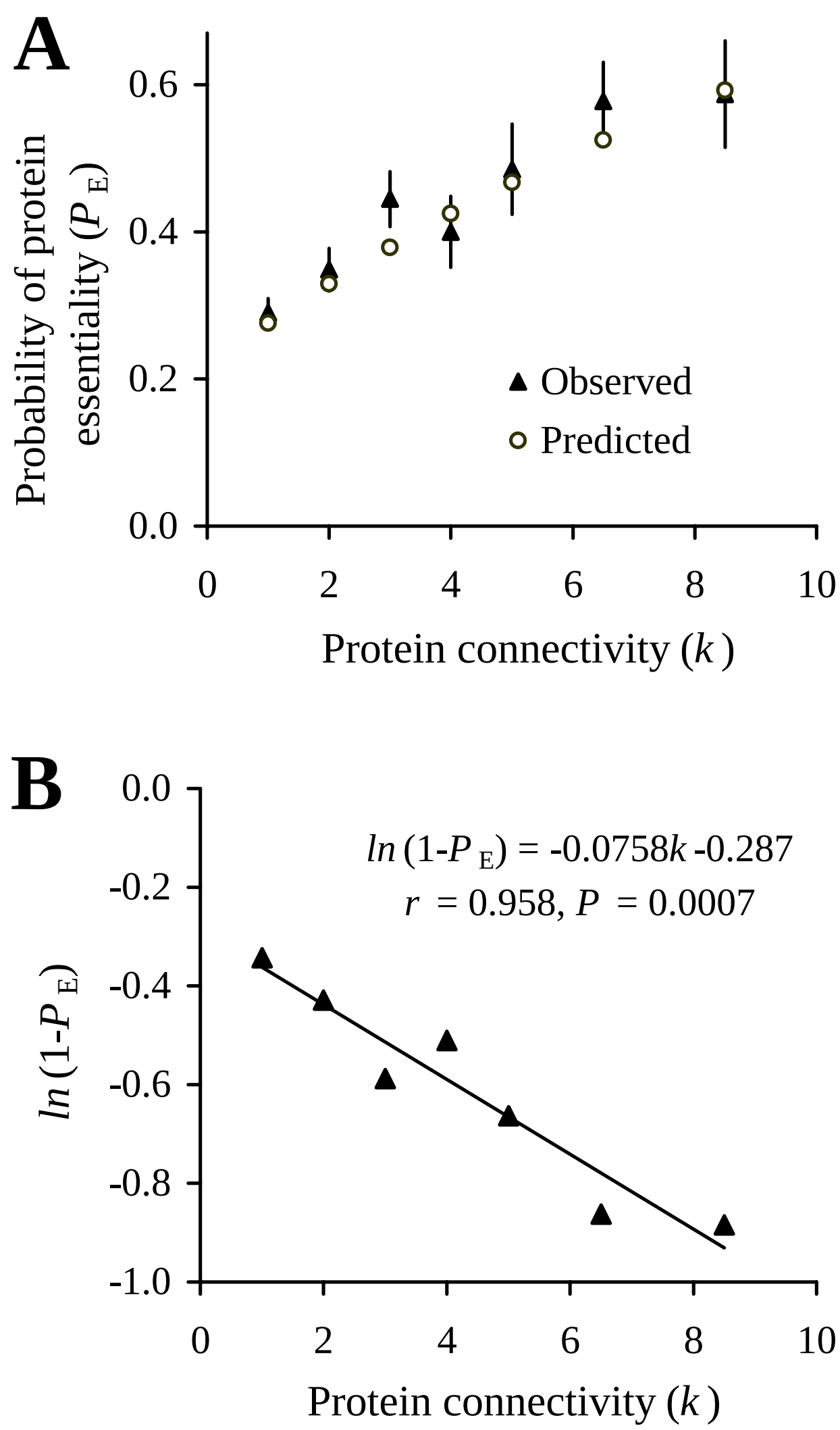
A
0.6
0.4
0.2
0.0
Probability of protein essentiality (P E)
Observed
Predicted
0 2 4 6 8 10
Protein connectivity (k)
B
0.0
-0.2
-0.4
-0.6
-0.8
-1.0
ln (1-P E)
ln (1-P E) = -0.0758k -0.287
r = 0.958, P = 0.0007
0 2 4 6 8 10
Protein connectivity (k)

Figure S2
