# Supplementary material for: Why Do Hubs Tend to Be Essential in Protein Networks?
Source: PLoS Genet. 2006 Jun 2;2(6):e88. doi: 10.1371/journal.pgen.0020088 (PMC1473040; doi:10.1371/journal.pgen.0020088)
Supplement: Supplementary file 3 [file pgen.0020088.sg003.pdf]

A

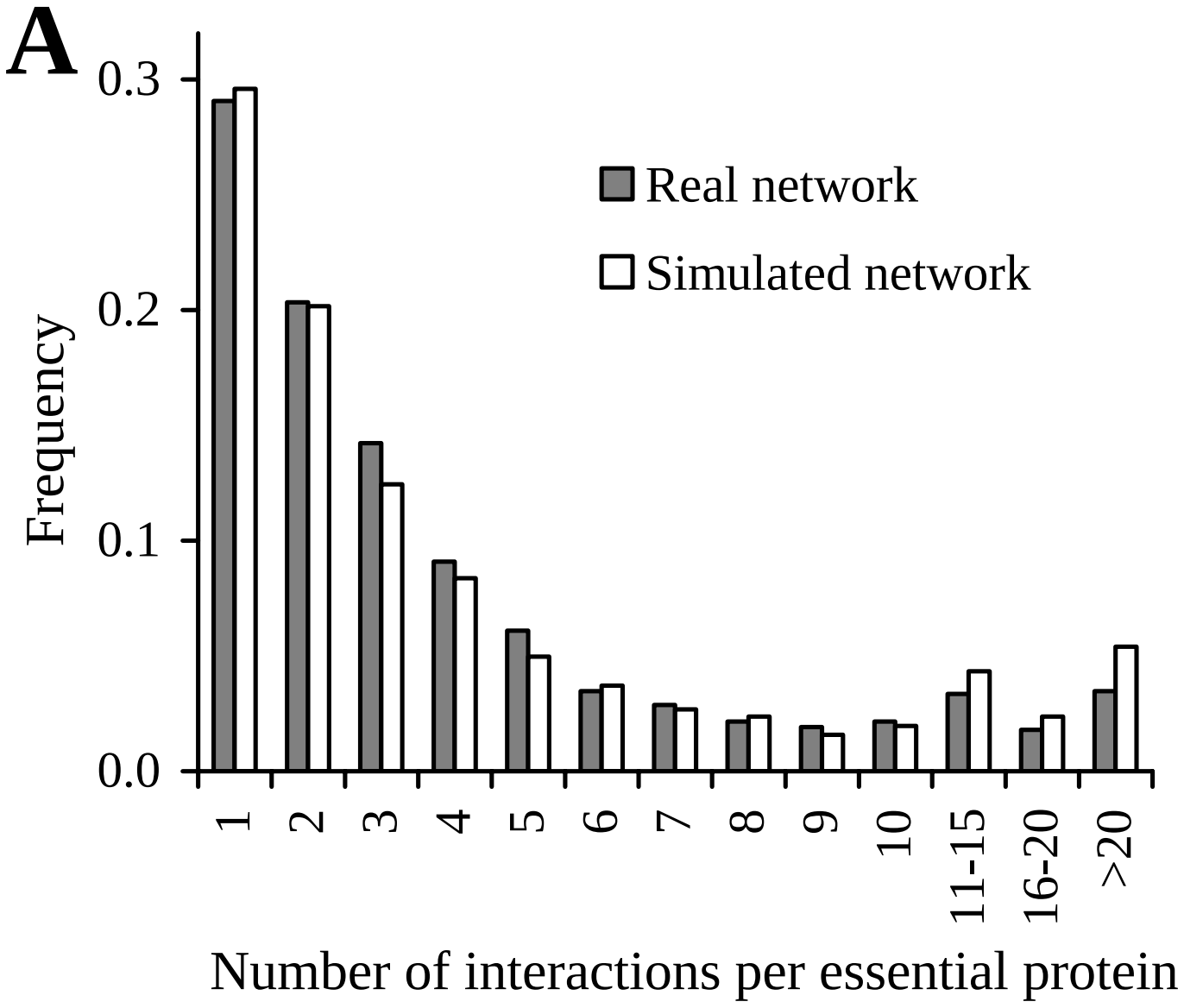

0.3
0.2
0.1
0.0
Frequency
Real network
Simulated network
1
2
3
4
5
6
7
8
9
10
11-15
16-20
>20
Number of interactions per essential protein

B

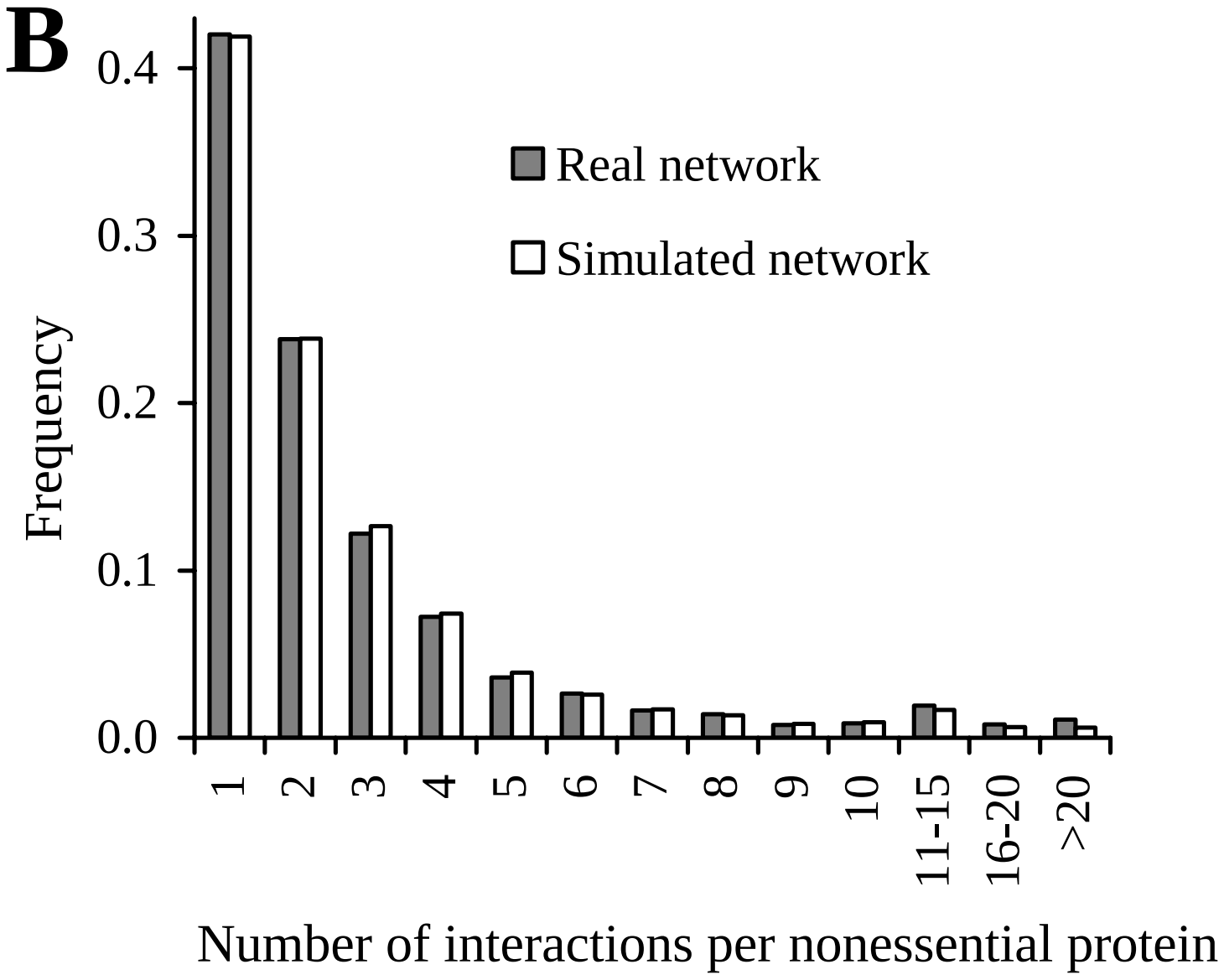

0.4
0.3
0.2
0.1
0.0
Frequency
Real network
Simulated network
1
2
3
4
5
6
7
8
9
10
11-15
16-20
>20
Number of interactions per nonessential protein

Figure S3
