# Supplementary material for: Why Do Hubs Tend to Be Essential in Protein Networks?
Source: PLoS Genet. 2006 Jun 2;2(6):e88. doi: 10.1371/journal.pgen.0020088 (PMC1473040; doi:10.1371/journal.pgen.0020088)
Supplement: Supplementary file 5 [file pgen.0020088.sg005.pdf]

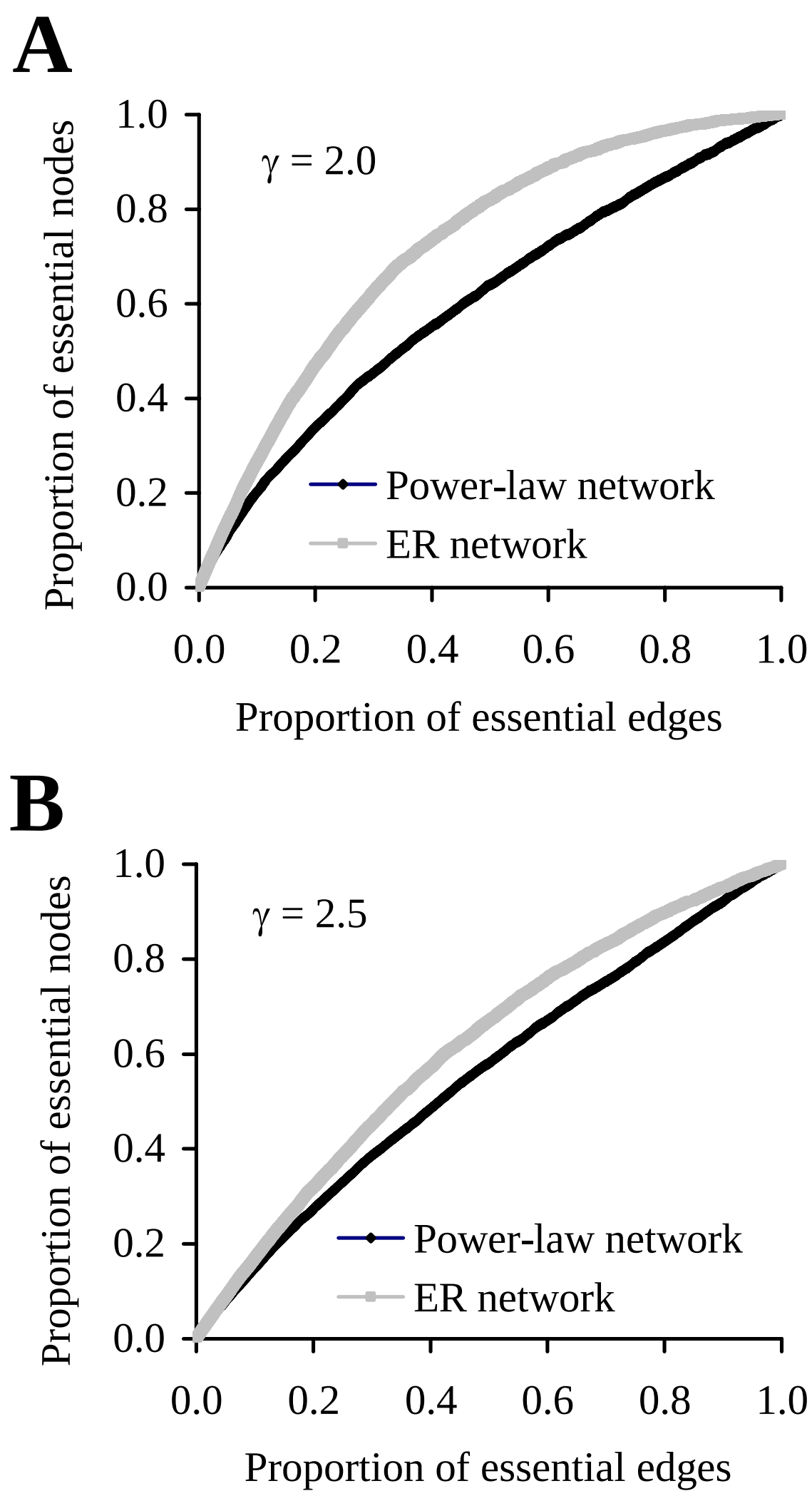

A
γ = 2.0
Proportion of essential nodes
1.0
0.8
0.6
0.4
0.2
0.0
Power-law network
ER network
0.0
0.2
0.4
0.6
0.8
1.0
Proportion of essential edges
B
γ = 2.5
Proportion of essential nodes
1.0
0.8
0.6
0.4
0.2
0.0
Power-law network
ER network
0.0
0.2
0.4
0.6
0.8
1.0
Proportion of essential edges

Figure S5
