# Supplementary material for: Why Do Hubs Tend to Be Essential in Protein Networks?
Source: PLoS Genet. 2006 Jun 2;2(6):e88. doi: 10.1371/journal.pgen.0020088 (PMC1473040; doi:10.1371/journal.pgen.0020088)
Supplement: Supplementary file 6 [file pgen.0020088.st001.pdf]

Table S1. Probably essential PPIs in the yeast.

Gene 1			Gene 2		
Gene ID	Gene name[1]	Gene function	Gene ID	Gene name	Gene function
YPL020C	ULP1	Ubl-specific protease	YBR038W	CHS2	Chitin synthase II
YJL018W	MPS3	Component of the yeast spindle pole body	YBR070C	ALG14	Essential protein required for the second step of dolichyl-linked oligosaccharide synthesis
YFL022C	FRS2	Phenylalanine--tRNA ligase alpha chain, cytosolic	YDL193W	NUS1	Putative nuclear undecaprenyl pyrophosphate synthase
YML130C	ERO1	Protein required for protein disulfide bond formation in the ER	YDR021W	FAL1	Protein involved in maturation of 18S rRNA
YOR260W	GCD1	Translation initiation factor eIF2bgamma subunit	YDR211W	GCD6	Translation initiation factor eIF2b epsilon, 81 kDa subunit
YAL001C	TFC3	TFIIIC (transcription initiation factor) subunit, 138 kD	YDR362C	TFC6	TFIIIC (transcription initiation factor) subunit, 91 kD
YHR065C	RRP3	Protein required for maturation of the 35S primary transcript	YDR367W	?	Protein of unknown function localised to golgi-ER transport vesicles
YCR093W	CDC39	Nuclear protein	YDR376W	ARH1	Mitochondrial protein putative ferredoxin-NADP+ reductase
YBR136W	MEC1	Cell cycle checkpoint protein	YDR499W	LCD1	Cell cycle checkpoint protein
YPR108W	RPN7	Subunit of the regulatory particle of the proteasome	YER021W	RPN3	26S proteasome regulatory subunit
YDR397C	NCB2	Negative Cofactor B2 is the beta subunit of a negative regulator of RNA polymerase II holoenzyme	YER159C	BUR6	Functional homolog of human NC2alpha
YDR238C	SEC26	Coatomer complex beta chain of secretory pathway vesicles	YFR051C	RET2	Coatomer complex delta chain
YDR113C	PDS1	Cell cycle regulator	YGR098C	ESP1	Protein required for sister chromatid separation
YMR260C	TIF11	Translation initiation factor eIF1a	YGR156W	PTI1	Pta1p Interacting protein
YDR267C	CIA1	Essential protein involved in assembly of cytosolic and nuclear iron-sulfur proteins	YHR122W	?	Protein of unknown function localised to cytoplasm
YDR212W	TCP1	Component of chaperonin-containing T-complex	YIL142W	CCT2	Chaperonin of the TCP1 ring complex, cytosolic
YDL235C	YPD1	Phosphorelay intermediate between Sln1p and Ssk1p	YIL147C	SLN1	Two-component signal transducer

YLR409C	UTP21	Possible U3 snoRNP protein involved in maturation of pre-18S rRNA	YJL069C	UTP18	Possible U3 snoRNP protein involved in maturation of pre-18S rRNA
YDL102W	CDC2	DNA-directed DNA polymerase delta, catalytic 125 KD subunit	YJR006W	HYS2	DNA-directed DNA polymerase delta, 55 KD subunit
YBR087W	RFC5	DNA replication factor C, 40 KD subunit	YJR068W	RFC2	DNA replication factor C, 41 KD subunit
YGL155W	CDC43	Geranylgeranyltransferase beta subunit	YKL019W	RAM2	Protein farnesyltransferase, alpha subunit
YJL090C	DPB11	Protein involved in DNA replication and S-phase checkpoint	YKL108W	SLD2	Chromosomal DNA replication protein
YDL092W	SRP14	Signal recognition particle subunit	YKL122C	SRP21	Signal recognition particle subunit
YDR292C	SRP101	Signal recognition particle receptor, alpha chain	YKL154W	SRP102	Signal recognition particle receptor, beta chain
YDL150W	RPC53	DNA-directed RNA polymerase III, 47 KD subunit	YKR025W	RPC37	Pol III transcription
YOR282W	?	Unknown protein	YLL018C	DPS1	Aspartyl-tRNA synthetase, cytosolic
YHL015W	RPS20	Ribosomal protein	YLL031C	GPI13	Protein involved in glycosylphosphatidylinositol biosynthesis
YIR012W	SQT1	Suppresses dominant-negative mutants of the ribosomal protein QSR1	YLR075W	RPL10	60S large subunit ribosomal protein
YBR190W	?	Unknown protein	YLR117C	CLF1	Pre-mRNA splicing factor
YDR086C	SSS1	ER protein-translocase complex subunit	YLR378C	SEC61	ER protein-translocation complex subunit
YER038C	KRE29	Killer toxin REsistant protein	YML023C	NSE5	Essential subunit of the Mms21-Smc5-Smc6 complex involved in DNA repair
YFL037W	TUB2	Beta-tubulin	YML085C	TUB1	Alpha-1 tubulin
YDR478W	SNM1	RNA binding protein of RNase MRP	YNL221C	POP1	Protein component of ribonuclease P and ribonuclease MRP
YOL094C	RFC4	DNA replication factor C, 37 kDa subunit	YNL290W	RFC3	DNA replication factor C, 40 kDa subunit
YJL034W	KAR2	Nuclear fusion protein	YOR254C	SEC63	ER protein-translocation complex subunit
YDR190C	RVB1	RUVB-like protein	YPL235W	RVB2	RUVB-like protein
YPR094W	RDS3	Protein required for cell viability	YPR133C	SPN1	Protein is Spt6-interacting putative elongation factor
YJL031C	BET4	Geranylgeranyl transferase, alpha chain	YPR176C	BET2	Geranylgeranyltransferase type II beta subunit

[1] Genes without names are indicated by a question mark.
