# Supplementary material for: Why Do Hubs Tend to Be Essential in Protein Networks?
Source: PLoS Genet. 2006 Jun 2;2(6):e88. doi: 10.1371/journal.pgen.0020088 (PMC1473040; doi:10.1371/journal.pgen.0020088)
Supplement: Supplementary file 7 [file pgen.0020088.st002.pdf]

Table S2. Conserved PPIs between the yeast and fruitfly.

Yeast gene 1	Yeast gene 2	Fruitfly gene 1	Fruitfly gene 2
YBL026W	YJL124C	CG10418	CG4279
YIL034C	YKL007W	CG17158	CG10540
YGL094C	YKL025C	CG8232	CG11486
YIL103W	YKL191W	CG11652	CG7265
YGR163W	YML121W	CG8707	CG11968
YER112W	YNL147W	CG31990	CG13277
YER146W	YNL147W	CG6610	CG13277
YBL050W	YOL018C	CG6625	CG1467
YGL095C	YOL018C	CG8228	CG1467
YJR102C	YPL002C	CG14750	CG6637
YHL002W	YNR006W	CG6521	CG2903
YGL237C	YOR358W	CG3891	CG3075
YFL039C	YNL138W	CG4027	CG5061
YDL081C	YLR340W	CG4087	CG7490
YBR170C	YGR048W	CG4673	CG6233
YHR012W	YJL154C	CG4764	CG5625
YLR170C	YPR029C	CG5864	CG9113
YCL055W	YGL192W	CG7818	CG5933
YLR200W	YML094W	CG7770	CG7048
YKL041W	YLR025W	CG9779	CG8055
**YBR254C**	**YDR472W**	**CG5161**	**CG10153**
**YJR072C**	**YOR262W**	**CG3704**	**CG10222**
**YGR083C**	**YLR291C**	**CG10315**	**CG2677**
**YNL113W**	**YPR110C**	**CG10685**	**CG3756**
**YJR093C**	**YPR107C**	**CG1078**	**CG3642**
**YDR299W**	**YGR145W**	**CG11188**	**CG7516**
**YJR002W**	**YNL075W**	**CG13097**	**CG11920**
**YGR172C**	**YNL263C**	**CG12404**	**CG5484**
**YLR275W**	**YPR182W**	**CG1249**	**CG16792**
**YER146W**	**YLR275W**	**CG6610**	**CG1249**
**YHR148W**	**YJR002W**	**CG4866**	**CG13097**
**YIL106W**	**YNL161W**	**CG13852**	**CG8637**
**YDR489W**	**YJL072C**	**CG14549**	**CG18013**
**YDR013W**	**YDR489W**	**CG9187**	**CG14549**
**YOR159C**	**YPR182W**	**CG18591**	**CG16792**
**YGL044C**	**YMR061W**	**CG7697**	**CG17170**
**YMR309C**	**YNL244C**	**CG4954**	**CG17737**
**YFR004W**	**YOR261C**	**CG18174**	**CG3416**
**YMR240C**	**YOR319W**	**CG3605**	**CG3780**
**YDL195W**	**YLR208W**	**CG8266**	**CG6773**
**YIL021W**	**YOL005C**	**CG7885**	**CG6840**
YDR397C	***YER159C***	***CG4185***	***CG10318***
YJL031C	***YPR176C***	***CG12007***	***CG18627***
YDR267C	***YHR122W***	***CG12797***	***CG7949***
YNL290W	***YOL094C***	***CG5313***	***CG14999***
YDR190C	***YPL235W***	***CG4003***	***CG9750***

Yeast IBEPs are boldfaced and probably essnetial PPIs are in italics.
